# *Streptomyces chodovensis* sp. nov.: a novel candidate for plant growth-promoting actinomycete from bare soil developing on Miocene lacustrine sediment of the Sokolov Coal Basin, Czech Republic

**DOI:** 10.1007/s10482-026-02440-5

**Published:** 2026-10-17

**Authors:** Alper Dede, Vaclav Krištůfek, Lucie Kotrbová, Kateřina Petříčková, Terézia Horváthová, Christopher M. Field, Sarah Kirstein, Meina Neumann-Schaal, Imen Nouioui, Alica Chroňáková

**Affiliations:** 1 Biology Centre Czech Academy of Sciences, Institute of Soil Biology and Biogeochemistry, Na Sádkách 702/7, 37005 České Budějovice, Czech Republic; 2 Biology Centre Czech Academy of Sciences, Institute of Parasitology, Branišovská 1160/31, 370 05 České Budějovice, Czech Republic; 3 Faculty of Science, University of South Bohemia, Branišovská, 37005 České Budějovice, Czech Republic; 4 Institute of Microbiology and Immunology, First Faculty of Medicine, Charles University, Prague, Czech Republic; 5 Institute of Microbiology, ETH Zurich, Vladimir-Prelog-Weg 4, 8093 Zurich, Switzerland; 6 Leibniz Institute DSMZ - German Collection of Microorganisms and Cell Cultures, Inhoffenstraße 7B, 38124 Brunswick, Germany; 7 Present Address: University Hospital Basel, Petersgraben 4/Spitalstrasse 21, 4031 Basel, Switzerland

**Keywords:** Actinobacteria, Plant growth-promoting traits, Polyphasic taxonomy, Post-mining sites, *Streptomyce*s

## Abstract

A novel actinomycete, designated as BCCO 10_0861^T^, was isolated from overburden Miocene clay sediments undergoing primary succession in Northwest Czechia and characterized by the polyphasic approach. According to 16S rRNA gene sequence similarity, the strain was closely related to *Streptomyces turgidiscabies* DSM 41838^T^ (99.00%), *Streptomyces reticuliscabiei* DSM 41804^T^ (98.93%), *Streptomyces lacrimifluminis* DSM 111125^T^ (98.15%), and *Streptomyces geranii* JCM 32177^T^ (98.08%). Morphological and chemotaxonomic characteristics supported the assignment to the genus *Streptomyces*. Diagnostic diamino acid in the cell-wall peptidoglycan was *LL*-diaminopimelic acid and the dominant menaquinone was MK-9(H_6_). The major cellular fatty acids (> 10%) were *anteiso*-C_15:0_, *iso*-C_16:0_, C_16:1_ ω7c, and C_16:0_. The polar lipid profile comprised of aminophospholipids, diphosphatidylglycerol, glycophospholipids, phosphatidylethanolamine, phosphatidylinositol, and three unidentified phospholipids. The whole cell sugars were galactose, glucose, mannose, ribose, and traces of rhamnose. The genome size of the strain BCCO 10_0861^T^ was 10.6 Mb with G+C content of 70.05%. Bioinformatic analysis suggested several biosynthetic gene clusters (BGCs) with medium-to-high similarity confidence to already known BGCs, having ethylenediaminesuccinic acid hydroxyarginine (EDHA), humidimycin, inthomycin B, and triascin C unique to BCCO 10_0861^T^. Similarly, predicted plant-growth promoting trait (PGPT) genes differed between genomes, when the one of BCCO 10_0861^T^ showed the highest number of unique PGPT genes (2,255). In addition, no association with potato pathogenicity was predicted in the genome of BCCO 10_0861^T^. The digital DNA-DNA hybridization (ranged from 34.7 to 37.5%), average nucleotide identity values (ranged from 86.26 to 87.24%) and phenotype distinguish strain BCCO 10_0861^T^ from closely related strains. Based on the polyphasic approach and genomic content, the strain represents a novel species of the genus** Streptomyces**, for which we propose the name** Streptomyces chodovensis** sp. nov. for BCCO 10_0861^T^ (= DSM 117345^T^).

**Supplementary Information:**

The online version contains supplementary material available at https://doi.org/10.1007/s10482-026-02440-5.

## Highlights

- Streptomyces strain was isolated from early successional stage on Miocene clay
- Phylogeny showed a close relationship to plant endophytes or pathogens
- Genomic analysis excluded pathogenicity island related to potato scab pathogens
- Strain BCCO 10_0861^T^ showed weak antibacterial and moderate antifungal activities
- Strain showed some plant growth promoting traits, fewer than predicted by genomic analysis

**Supplementary Information:**

The online version contains supplementary material available at https://doi.org/10.1007/s10482-026-02440-5.

## Introduction

Streptomycetes are aerobic, Gram-stain-positive actinobacteria with a high DNA G+C content, able to form branched substrate and aerial mycelia that differentiate into chains of few to many spores (Kämpfer et al. 2014). In addition, they are characterized to have *LL*-diaminopimelic acid (*LL*-DAP) and no diagnostic sugars in whole-cell hydrolysates (Waksman and Henrici 1943; Skerman et al. 1980; Anderson and Wellington 2001). As a member of Terrabacteria clade, streptomycetes are associated with an early colonization of land that occurred between 2.5 to 3.2 billion years ago (Battistuzzi et al. 2004), evolving in tight connection with plants and plant-derived organic debris (Chater 2016; Viaene et al. 2016; Chevrette et al. 2019). Many of them are considered plant growth-promoting bacteria abundant in the rhizosphere (Dede et al. 2020; Nicolle et al. 2024) or endophytic compartments (Dede and Güven 2023), while only a few species act as plant pathogens, like *Streptomyces acidiscabies*, *Streptomyces europaescabiei*, *Streptomyces turgidiscabies*, and *Streptomyces scabies* (Gutierrez et al. 2022). However, *Streptomyces* spp. inhabit widespread habitats in a global ecosystem: soil, freshwater, marine, and air as free-living or in association with plants, invertebrates, and mammals (Li et al. 2019).

*Streptomyces* spp. form the largest prokaryotic genus (Waksman and Henrici 1943), 811 validly published species with correct names (LPSN website https://lpsn.dsmz.de/genus/streptomyces, accessed June 10th, 2026). Genetic diversity can be attributed to the enormous genome plasticity enabled by large linear chromosome (4.8–13.6 Mb), comprising central core genome encompassing essential genes and dispensable, fast-evolving ends (arms). These terminal regions are prone to gene exchange through lateral gene transfer and contribute to their adaptability to fluctuating environmental conditions and ability to colonize novel niches (Almeida et al. 2019). The interest in discovering and describing new species in this genus is not waning, but rather the opposite, as demonstrated by Komaki (Komaki 2023). This trend is undoubtedly due to recent progress in whole genome sequencing-based methods used in taxonomic classification (Nouioui et al. 2018), or genome-mining technologies as; antiSMASH, MiBiG, ARTS, or others (Wattam et al. 2017; Patz et al. 2021; Mungan et al. 2022; Blin et al. 2025; Zdouc et al. 2025), but also to their cultivability and the recent exploration of new environments, such as marine, underground, or host-associated habitats (Wang et al. 2022). However, the high plasticity of their genome (Zhou et al. 2012), which allows them a multitude of metabolic adaptations to the environmental changes (Li et al. 2019), including a very diverse secondary metabolism, is certainly another driving force behind this trend (Caicedo-Montoya et al. 2021; Otani et al. 2022; Nikolaidis et al. 2023; Jørgensen et al. 2024).

One of the unexplored ecosystems is of anthropogenic origin: bare soils developing after open cast mining. These soils provide a unique opportunity to study ecological succession and microbial colonization in environments drastically altered by human activity. Surface mining of lignite affects large areas as it exposes and brings to the surface the geological strata overlying the lignite seam (Kříbek et al. 1998). In Velká Podkrušnohorská spoil heap (Sokolov brown coal mining area, Czech Republic), many Miocene clays have been overburdened in this way, which have then been used for reclamation or left for primary succession (Helingerová et al. 2010). These clays (cypris) are highly specialized habitats that harbor for taxonomically distinct and diverse microorganisms, and can serve as reservoirs for yet unexplored microbial diversity (Elhottová et al. 2006; Chroňáková et al. 2010). Actinomycetes isolated from Miocene clays and different successional stages were the basis for the establishment of the Culture Collection of Actinomycetes at BCCO (www.actinomycetes.bcco.cz), where more than 600 isolates originate from this area. Using polyphasic approaches, we recently described three new species of the genus *Lentzea* (Lara et al. 2024).

Strain BCCO 10_0861^T ^= DSM 117345^T^ was initially isolated for bioprospecting purposes, subsequent phenotypic profiling revealed a distinct combination of aerial mycelium coloration and soluble pigment production that distinguished it from other isolates. Subsequent whole-genome sequencing and comprehensive comparative genomic analyses confirmed its distinct phylogenetic position. Consequently, based on robust polyphasic evidence, we propose a novel species represented by this single isolate. The strain was characterized in comparison with its closest phylogenetic relatives: *Streptomyces geranii* JCM 32177^T^, *Streptomyces lacrimifluminis* DSM 111125^T^, *Streptomyces turgidiscabies* DSM 41838^T^, and *Streptomyces reticuliscabiei* DSM 41804^T^. *S. geranii* is a plant endophyte (Li et al. 2018), while *S. turgidiscabies*, and *S. reticuliscabiei* are reported as plant pathogens causing raised or netted scab lesions on potato tuber (Miyajima et al. 1998; Bouchek-Mechiche et al. 2000). This study expands the taxonomic framework of *Streptomyces* by integrating genomic and phenotypic data from an anthropogenic habitat. Our findings highlight the adaptability of the genus and underscore the potential of disturbed ecosystems as reservoirs of microbial novelty.

## Identification and phylogenetic analysis using 16S rRNA gene and whole genome sequences

Strain BCCO 10_0861^T^ was isolated from soil at the top of reclaimed mine heaps in the early successional stage in the Sokolov brown coal mine district area in the Czech Republic (50.262 N, 12.731 E). The strain was isolated from bare soil developed on Miocene lacustrine clay (pH 7.8) deposited after brown coal mining in 2008 on Reasoner’s 2A (R2A) agar (HiMedia Laboratories Pvt. Ltd., Mumbai, India). Cultures were maintained on yeast extract–malt extract agar (International *Streptomyces* Project (ISP) 2 medium) (Shirling and Gottlieb 1966) and as cell suspensions in 15% (v/v) glycerol stocks at −76 °C for long-term preservation. Genomic DNA of BCCO 10_0861^T^ was extracted from a three-day-old bacterial culture grown in Luria–Bertani (LB) broth at 28 °C (± 2 °C) with shaking at 150 rpm (Bertani 1951). The NucleoSpin Microbial DNA Kit (Macherey–Nagel) was used for DNA extraction, following the manufacturer’s instructions. The DNA quantity and quality were analyzed using a NanoDrop 2000 spectrophotometer and agarose gel electrophoresis. The almost complete 16S rRNA gene was amplified by PCR using the bacterial universal primers pA (5´-AGAGTTTGATCCTGGCTCAG-3´) and pH (5´-AAGGAGGTGATCCAGCCGCA-3´) (Edwards et al. 1989). The purified PCR products were bidirectionally sequenced in Seqme, s.r.o., Dobříš, Czech Republic. The sequences were assembled with BioEdit (Hall 1999). The 16S rRNA genes of the closest type species were retrieved from EzBioCloud database (Kim et al. 2012). According to EzBioCloud database (updated on April, 21st, 2025), 16S rRNA gene sequence comparisons showed that strain BCCO 10_0861^T^ has a similarity of 99.00% to *S. turgidiscabies* DSM 41838^T^ (14 bp difference), 98.93% to *S. reticuliscabiei* DSM 41804^T^ (15 bp difference), 98.65% to *S. humidus* NBRC 12877^T^ (19 bp difference), and 98.29% to *S. asoensis* NRRL B-16592^T^ (24 bp difference), respectively.

The evolutionary history of BCCO 10_0861^T^ was initially constructed using multiple tree-building algorithms to ensure topological robustness. Briefly, the sequence alignments were aligned with CLUSTALW on MEGA 11 (Edgar 2004; Tamura et al. 2021). A Maximum-Likelihood (ML) tree was inferred based on the Tamura-Nei substitution model (Tamura and Nei 1993). Parallel evolutionary distance evaluations and tree constructions were executed via the Neighbor-Joining (NJ) method using Jukes–Cantor evolutionary distances (Jukes and Cantor 1969). The percentage of replicate trees in which the associated taxa clustered together was calculated in the bootstrap test (1,000 replicates) (Felsenstein 1985).

**Fig. 1:**
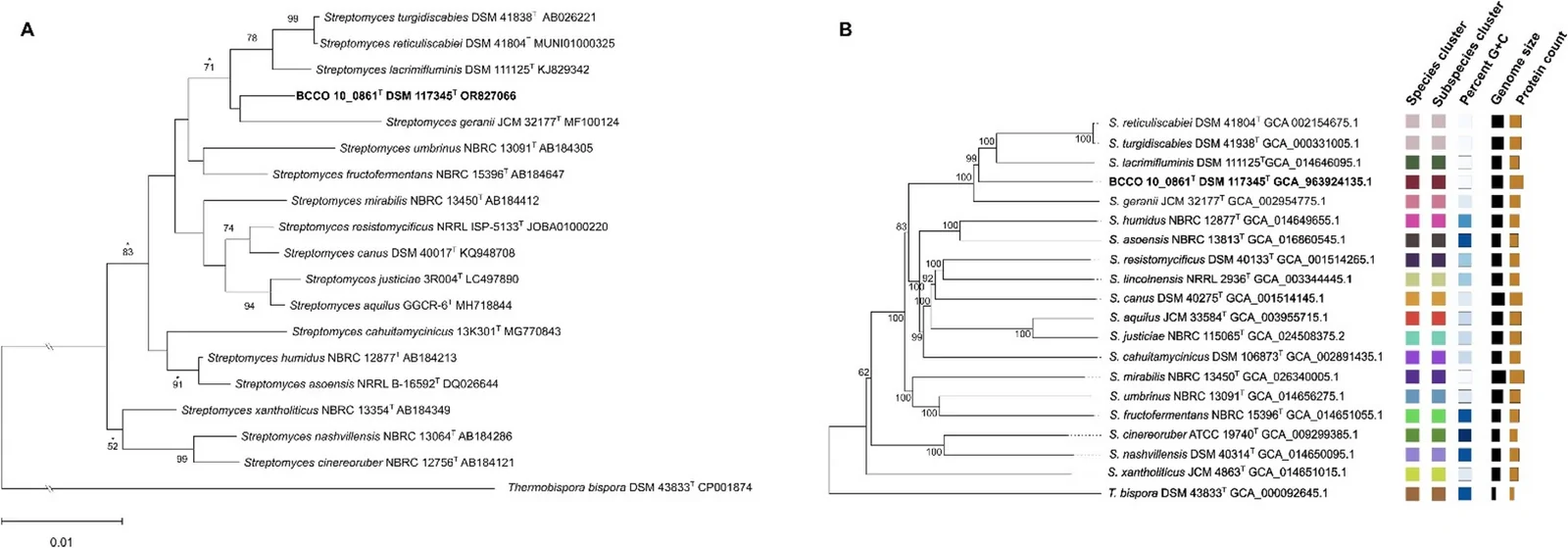
Phylogenetic and genomic analysis of BCCO 10_0861^T^ and the closest *Streptomyces* strains. **A** Maximum likelihood tree illustrating the phylogenetic relationships, with BCCO 10_0861^T^ showing closest similarity to *S. geranii* JCM 32177^T^, *S. lacrimifluminis* DSM 111125^T^, *S. turgidiscabies* DSM 41938^T^, and *S. reticuliscabiei* DSM 41804^T^. Nodes also supported by neighbor-joining (Supplementary Fig 2) analysis are marked with an asterisk (*). Bootstrap values are based on 1,000 replicates. **B** Genome-based phylogeny inferred from the TYGS database, highlighting the proximity of BCCO 10_0861^T^ to *S. lacrimifluminis* DSM 111125^T^, *S. reticuliscabiei* DSM 41804^T^, *S. turgidiscabies* DSM 41938^T^, and *S. geranii* JCM 32177^T^. The tree includes annotations for species clusters, G+C content, genome size, and protein count. Branch support was inferred from 100 pseudo-bootstrap replicates. Both trees are using *Thermobispora bispora* DSM 43833^T^ as an outgroup

The ML topology (Fig. 1A) and the corresponding NJ tree (Supplementary Fig 1) revealed that strain BCCO 10_0861^T^ forms a highly stable, distinct monophyletic lineage clusters alongside well-defined reference strains. Both algorithms independently confirmed that the phylogenetically closest type strains to the BCCO 10_0861^T^ are *S. geranii* JCM 32177^T^, *S. lacrimifluminis* DSM 111125^T^, *S. turgidiscabies* DSM 41838^T^, and *S. reticuliscabiei* DSM 41804^T^, respectively. Node stability was strongly supported by high bootstrap values (71–99%) and critical nodes shared across both the ML and NJ analyses were marked with the asterisk (*), verifying the consistency of the sequence-based datasets.

## Genome features

Genome sequencing was performed at the Institute of Microbiology (ETH Zurich) on a MinION with an R10.4.1 flow cell and base called with the model dna_r10.4.1_e8.2_260bps_hac@v3.5.2. In total, there were 96,726 reads with a median length of 1,377 bp and median quality score of 12.0, summing to 244.6 Mbp. Reads were filtered for a minimum length of 1,000 bp with Filtlong (v0.2.1; https://github.com/rrwick/Filtlong). The genome was assembled with Flye (v2.9; https://doi.org/10.1038/s41587-019-0072-8) using standard parameters, resulting in a genome size of 10,631,911 base pairs, assembled in 11 contigs, with an N50 of 182.7 Kb (Table 1). The reads and assembly were deposited at the ENA under the study accession ERP155806, sample accession ERS17732811, reads accession ERR12495476, and assembly accession ERZ22297350. The quality of the reads was evaluated using fastQC (Andrews 2010), and sequences were trimmed using Trimmomatic version 0.36 (Bolger et al. 2014). Clean reads were assembled using SPAdes version 3.15.3 (Bankevich et al. 2012) and Velvet version 1.2.10 (Zerbino 2010) on the KBase platform (Arkin et al. 2018). The assembly of the genome was performed in PATRIC web-based tool (with the annotation option). Digital DNA-DNA hybridization (dDDH) was performed with TYGS genome server (Meier-Kolthoff and Göker 2019; Meier-Kolthoff et al. 2022). DNA G+C content was determined with PATRIC database (Olson et al. 2023). ANIb calculations were obtained from JSpeciesWS web-based tool. 16S rDNA sequences were retrieved from the genome with ContEst16S (Lee et al. 2017). The results from MinION and 16S rDNA sequence obtained by Sanger sequencing were compared, and no differences were observed.

**Table 1:** Genomic features of strain BCCO 10_0861^T^ and its close relatives

	1	2	3	4	5
Assembly ID	GCA_963924135.1	GCA_014646095	GCF_002154675.1	GCF_000331005.1	GCF_002954775
Genome size (bp)	10,631,911	8,982,869	10,825,282	9,962,914	9,002,516
Contigs	11	151	986	897	104
Contig N50 (kb)	182.7	247.2	22.8	27.4	175.5
Protein encoding genes (CDSs)	11,824	8,587	9,632	10,318	9,258
Cluster of orthologous groups (COG)	10,827	7,824	8,768	9,377	8,618
Plant-growth promoting traits (PGPT)	4,811	3,844	4,319	4,401	4,289
G+C content (mol%)	70.05	70	69.9	70.0	70.5
tRNA	68	77	77	78	69
rRNA	21	4	7	27	6

1- BCCO 10_0861^T^, 2- *S. lacrimifluminis* DSM 111125^T^, 3- *S. reticuliscabiei* DSM 41804^T^, 4- *S. turgidiscabies* DSM 41838^T^, 5- *S. geranii* JCM 32177^T^

BCCO 10_0861^T^ genome contained 11,824 protein-encoding genes (CDSs) and had a G+C content of 70.05% **(**Table 1**)**. To determine its genomic taxonomic position, whole-genome assembly of strain BCCO 10_0861^T^ was compared with 18 *Streptomyces* spp. type strains identified via the Type (Strain) Genome Server (TYGS) using *Thermobispora bispora* DSM 43833^T^ as an outgroup. Reference genome sequences were obtained from the NCBI genome database (www.ncbi.nlm.nih.gov/genome/), and the TYGS genome databases (Meier-Kolthoff and Göker 2019; Meier-Kolthoff et al. 2022). The Genome BLAST Distance Phylogeny (GBDP) method based on the pairwise intergenomic distances performed on TYGS database confirmed that the closest organisms to strain BCCO 10_0861^T^ are *S. lacrimifluminis* DSM 111125^T^, *S. reticuliscabiei* DSM 41804^T^, *S. turgidiscabies* DSM 41838^T^ and *S. geranii* JCM 32177^T^, respectively (Fig. 1B).

Genome-wide comparisons using TYGS and JSpeciesWS confirmed that strain BCCO 10_0861^T^ is distinct from its closest phylogenetic neighbors. The digital DNA-DNA hybridization (dDDH) values (d4) between BCCO 10_0861^T^ and closest *Streptomyces* species were significantly below the 70% threshold for species delimitation, ranging from 34.7 to 37.5%. Specifically, the highest similarity was observed with *S. lacrimifluminis* (37.5% dDDH; 0.04 G+C difference), followed by *S. reticuliscabiei* (36.7%), *S. turgidiscabies* (36.6%), and *S. geranii* (34.7%). Furthermore, Average Nucleotide Identity (ANIb) values across all analyzed strains ranged from 86.26 to 87.24%, below the 95%–96% species boundary (Supplementary Tables 1 and 2). These results collectively support the classification of BCCO 10_0861^T^ as a novel species within the genus *Streptomyces*.

## Strain ecology, morphological, biochemical, and physiological characteristics

The strain was deposited at the Culture Collection of Actinomycetes of the Biology Centre Collection of Organisms https://actinomycetes.bcco.cz/) under BCCO 10_0861^T^ and at Leibniz Institute DSMZ – German Collection of Microorganisms and Cell Cultures GmbH (www.dsmz.de/) under DSM 117345^T^. The close phylogenetic relative of strain BCCO 10_0861^T^, *S. lacrimifluminis* DSM 111125^T^, *S. turgidiscabies* DSM 41838^T^, and *S. reticuliscabiei* DSM 41804^T^, were obtained from the DSMZ collection (https://www.dsmz.de/collection/catalogue/microorganisms/catalogue) and included in the phenotypic analyses. *S. geranii* JCM 32177^T^ was not included in any of the laboratory experiments conducted in this study due to Nagoya protocol restrictions.

**Fig. 2:**
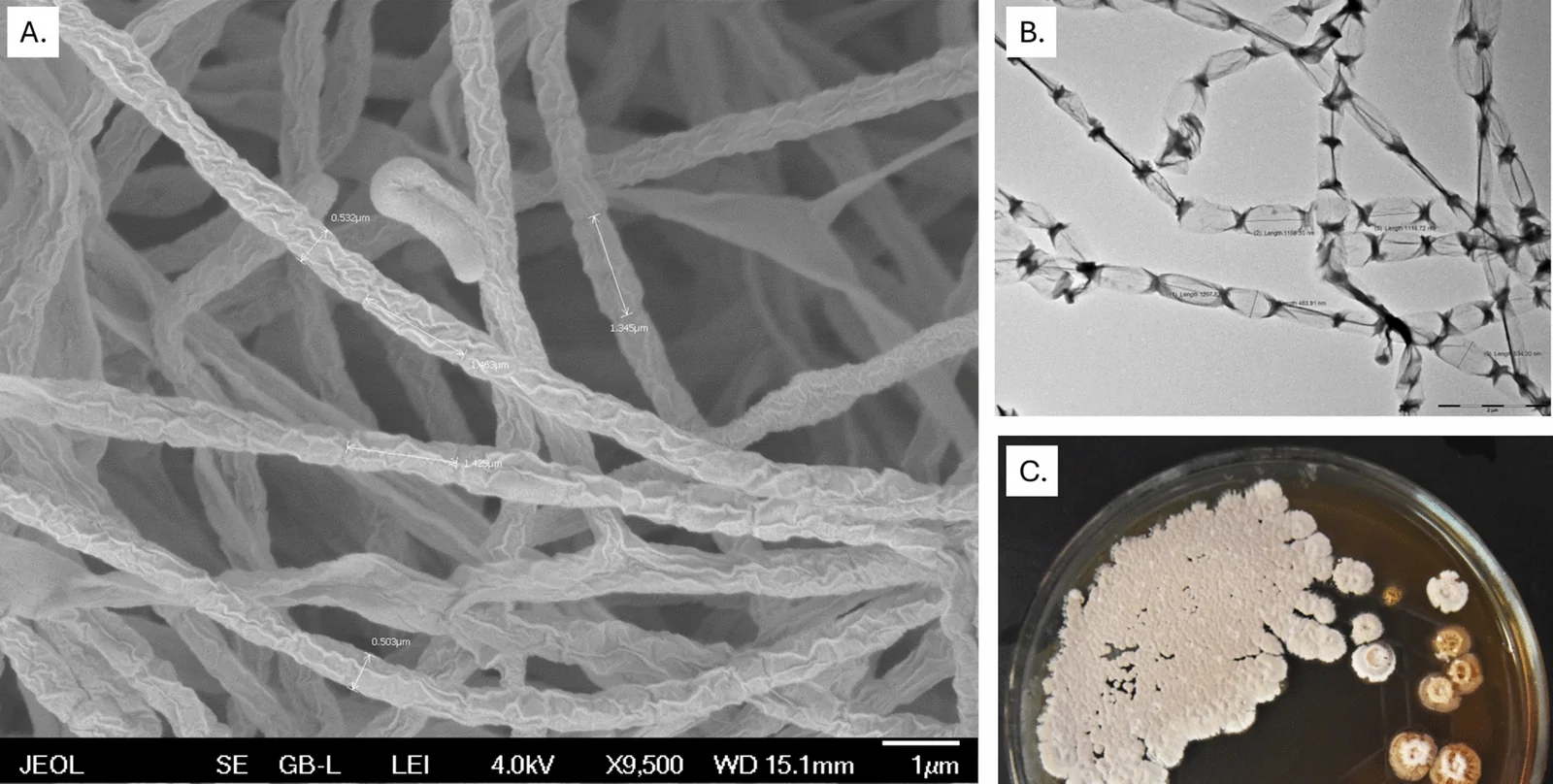
Morphological characteristics of BCCO 10_0861^T^ *(S. chodovensis* sp. nov.). **A** Scanning electron microscopy (SEM) image showing sporophore structure (rectus flexibilis type) of BCCO 10_0861^T^ after 21 days of incubation at 28 °C on ISP2 medium. **B** Transmission electron microscopy (TEM) image displaying spores with an average size of 1.464 µm × 0.682 µm (length × width). **C** Colony morphology of BCCO 10_0861^T^ on ISP2 medium, illustrating characteristic growth patterns after 21 days of incubation at 28 °C

Cultural characteristics were observed after growth at 28 °C for 2 weeks, using ISP2, ISP3, ISP4, ISP6 and ISP7 agar, and nutrient agar (Waksman 1940; Shirling and Gottlieb 1966). Colony colors were identified as described by Shirling and Gottlieb 1966. BCCO 10_0861^T^ with aerial spore mass of white color (White oyster, Wb) was developed well on ISP2, ISP4 media, and moderately on ISP3. Vegetative mycelium is pink-orange-brown and soluble pink-orange pigment is produced on ISP2, ISP3, ISP4 and ISP7 media. Cell morphology was examined using scanning electron microscopy (SEM) and transmission electron microscopy (TEM) following 21 days of incubation at 28 °C using ISP2 medium. BCCO 10_0861^T^ develops sporophores type rectus flexibilis, spores having a size of 1.464 µm × 0.682 µm (length x width, on average) (Fig. 2). Colony differences between BCCO 10_0861^T^ and strains representing the closest relative species are provided in Supplementary Table 3 and visualized on the ISP6 and ISP7 media in Supplementary Fig 2.

Strain BCCO 10_0861^T^ exhibits a mesophilic growth range of 4–30 °C, neutrophilic pH tolerance (6–8), and moderate halotolerance (0–2% NaCl) (Table 2). To ensure stability across the pH 4.0–12.0 range, a strategic multi-buffer system was implemented. Citrate–phosphate (pH 4–6), phosphate (pH 6–8), and carbonate-bicarbonate (pH 9–12) solutions were used to prevent unintended pH shifts (pH was measured in intervals of 1) (Beynon and Easterby 2004) and at temperatures of 4, 10, 20, 28, 30, 37, 45, and 55 °C with ISP2 medium. Additionally, sodium chloride (NaCl) tolerance was examined from 0 to 10% (in intervals of 1) using ISP2 medium. Other physiological characteristics, including the utilization of sole carbon sources were performed according to ISP project guidelines (Shirling and Gottlieb 1966; Wink 1990). Carbon utilization tests showed efficient assimilation of D-mannose, D-xylose, and glycerol (+ +), while D-ribose, cellulose, and D-fructose were not utilized (Table 2).

**Table 2:** Physiological, biochemical, and chemotaxonomic characteristics of strain—BCCO 10_0861^T^ and its close phylogenetic neighbors

	1	2*	3	4	5**
**Tolerance to**					
Temperature (°C)	4–30	10–37	25–41	25–30	10–37
pH (range 4–12)	6–8	6–11	4–11	5–11	6–11
NaCl (% 0–10)	0–2	0–4	0–2	0–2	0–5
**C utilization**					
D-mannitol	+	++	+	+	+
D-mannose	++	++	+	+	( +)
D-xylose	++	++	( +)	+	( +)
D-ribose	−	−	−	−	−
I-inositol	+	+	( +)	−	( +)
Sucrose	+	+	( +)	+	+
L-rhamnose	+	+	+	+	++
D-fructose	−	−	( +)	−−	−
L-arabinose	( +)	+	( +)	+	( +)
Glycerol	++	+	+	++	++
Cellulose	−	−	−	−	−
D-glucose	+	+	+	+	+
**Chemotaxonomy**					
Menaquinone	MK-9 (H_2_); 8.8%, MK-9 (H_4_); 14.0%, **MK-9 (H**_**6**_**); 57.6%,** MK-9 (H_8_); 19.6%	**MK-9 (H**_**6**_**); 48.9%,** **MK-9 (H**_**8**_**); 48.1%,** MK-8 (H_6_); 3%	MK-9 (H_2_); 2.3%, MK-9 (H_4_); 8.6%, **MK-9 (H**_**6**_**); 73.3%,** MK-9 (H_8_): 15.6%	MK-9 (H_2_); 0.7%, MK-9 (H_4_); 6.1%, **MK-9 (H**_**6**_**); 54.0%,** MK-9 (H_8_): 39.2%	MK-9 (H_4_)_;_ 16.7%, **MK-9 (H**_**6**_**)**_**;**_** 44.9%, MK-9( H**_**8**_**)**_**;**_** 38.4%**
Diaminopimelic acid (DAP)	*LL*-DAP	*LL*-DAP	*LL*-DAP	*LL*-DAP	*LL*-DAP
Whole cell sugars	Gal, Glu, Man, Rib, Rham (traces)	Gal	Gal, Glu, Man, Ara, Xyl, Rib,	Gal, Glu, Man, Ara, Xyl, Rib,	Gal, Rib
Polar lipids	DPG, PL, PE, APL, PI, GPL	DPG, PE, PI, PL, PG	DPG, AL, PE, PI, GPL	DPG, PL, PE, APL, PI, GPL	DPG, PE, PI, PL, APL
Major Fatty acids (> 5%)	*iso*-C_15:0_, *anteiso*-C_15:0_, C_16:1_ ω7c, *iso*-C_16:0_, C_16:0_, *anteiso*-C_17:0_	*iso*-C_15:0_, *anteiso*-C_15:0_, C_16:1_ ω7c, *iso*- C_16:0_, C_16:0_, *anteiso*-C_17:0_	*iso*-C_14:0,_ *iso*-C_15:0_, *anteiso*-C_15:0_, C_16:1_ ω7c, *iso*-C_16:0_, C_16:0_, *anteiso-*C_17:0_	*iso*-C_14:0_, C_15:0_, *iso*-C_15:0_, *anteiso*-C_15:0_, C_16:1_ ω7c, *iso*- C_16:0_, C_16:0_	*anteiso*-C_15:0_, C_15:0_, C_16:1_ ω7c, *iso*-C_16:0_, C_16:0_, *anteiso*-C_17:0_

Bold highlights the major menaquinones (>50%) in the given cathegory

* polar lipids and menaquinone data were acquired previously (Zhang et al. 2016); ** data acquired previously (Li et al. 2018); Growth is not better than the negative control -; Growth better than the negative control but not like the positive control ( +); Growth like the positive control +; Growth better than the positive control ++

DPG, diphosphatidylglycerol; GPL, glycophospholipids; PE, phosphatidylethanolamine; PG, phosphatidylglycerol; PI, phosphatidylinositol; PL, phospholipids, AL – aminolipid, APL-aminophospholipid. Ara, arabinose; Gal, galactose; Glu, glucose; Man, mannose; Rib, ribose; Rham, rhamnose; Xyl xylose

1- BCCO 10_0861^T^, 2- *S. lacrimifluminis* DSM 111125^T^ (Zhang et al. 2016), 3- *S. reticuliscabiei* DSM 41804^T^, 4- *S. turgidiscabies* DSM 41838^T^, 5- *S. geranii* JCM 32177^T^ (Li et al. 2018)

Enzymatic and other metabolic activities were performed with API ZYM, API 20NE and API 50CH strips (bioMérieux) following the instructions of the manufacturer. BCCO 10_0861^T^ shows distinct biochemical profiles compared to *S. lacrimifluminis* DSM 111125^T^, *S. reticuliscabiei* DSM 41804^T^, and *S. turgidiscabies* DSM 41838^T^, based on API ZYM, API 20 NE, and API 50CH tests. Compared to *S. lacrimifluminis*, BCCO 10_0861^T^ shows weaker α-mannosidase and α-fucosidase activity (weak vs. positive) in API ZYM, positive nitrate reduction and negative urease in API 20 NE, and in API 50CH, it is negative for D-arabinose, positive for raffinose and 5-keto-gluconate, with variable results for galactose, amygdalin, arbutin, salicin, and esculin (V1 vs. V2). Against *S. reticuliscabiei*, BCCO 10_0861^T^ displays weaker α-chymotrypsin, β-glucuronidase, and α-mannosidase in API ZYM, negative urease, positive adipate, citrate, and maltose, assimilation in API 20 NE, and in API 50CH, positive salicin, negative trehalose, positive amygdalin, with differences in arbutin, D-arabinose, esculin, and 2-keto-gluconate. Versus *S. turgidiscabies*, BCCO 10_0861^T^ has weaker β-glucuronidase and α-mannosidase in API ZYM, positive mannose, mannitol, citrate, and adipate assimilation in API 20 NE, and in API 50CH, positive salicin and lactose, negative maltose, positive amygdalin, and variable arbutin, esculin, and 2-keto-gluconate (full lists are given in Supplementary Table 4).

## Inference of biosynthetic potential, antibiotic resistance determinants and mobile genetic elements

Biosynthetic gene clusters (BGCs) were monitored using antiSMASH (Blin et al. 2025). Antimicrobial resistance genes were acquired from PATRIC’s ‘comparative genome analysis’. Antimicrobial resistance genes were retrieved from PATRIC (Wattam et al. 2017; Olson et al. 2023) and ResFinder CGE v.4.1 (Bortolaia et al. 2020), and mobile genetic elements (MGE) were evaluated using MGEfinder v.1.0.3 (Durrant et al. 2020) with 80% sequence identity (Dede et al. 2025).

**Fig. 3:**
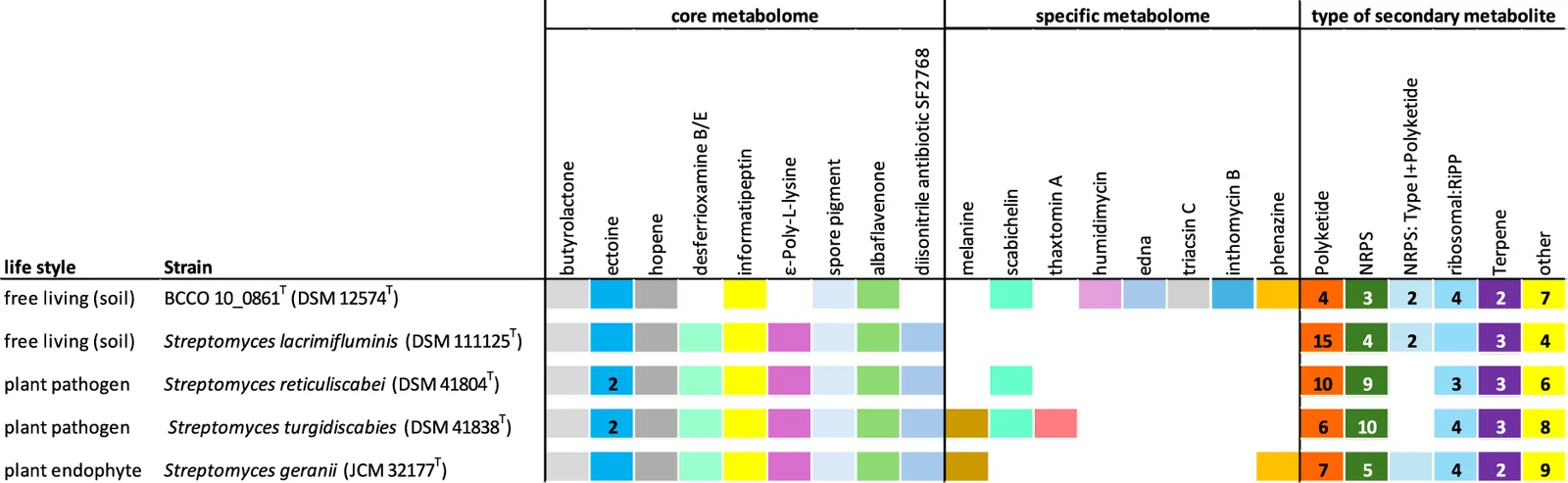
Schematic representation of core and strain-specific metabolome of BCCO 10_0861^T^ and the closest relatives as predicted by antiSMASH v7.0. Numbers indicate number of biosynthetic gene clusters predicted in the category. Complete list of predicted BGCs for all strains is given in Supplementary Table 5

Some of the BGCs usually found in majority of streptomycetes, representing the core metabolome (Vicente et al. 2018), were absent in strain BCCO 10_0861^T^: desferrioxamine B/E, diisonitrile antibiotic SF2768 and ε-Poly-L-lysine BGCs while they were identified in all other strains. BGCs present only in the genome of BCCO 10_0861^T^ were humidimycin, inthomycin B, triacsin C, and ethylenediaminesuccinic acid hydroxyarginine (EDHA), while scabichelin BGC was found in both plant-pathogenic species and phenazine in *S. geranii* JCM 32177^ T^ together with BCCO 10_0861^T^ (Fig. 3** and **Table 3). Both phenazines and scabichelin are considered specialized metabolites with different effects on plants: they can act as biocontrol agents or virulence factors and thus may have positive or negative effects on plants (Biessy and Filion 2018). Moreover, thaxtomin A BGC was only found in *S. turgidiscabies* DSM 41838^ T^. In summary, even the presence of BGC for putative virulence factors was recognized in the genome of BCCO 10_0861^T^, but only their concomitant and synergistic action may be associated with the plant virulence and pathogenicity (Polturak and Osbourni 2021). The complete list of biosynthetic gene clusters identified in all investigated strains is given in Supplementary Table 5.

**Table 3:** Secondary metabolite biosynthetic gene clusters across five *Streptomyces* strains. Similarity values to the closest known BGCs are presented

Secondary metabolite function	Most similar known cluster	1	2	3	4	5
Alkaloid+polyketide	Camporidine A/camporidine B	0	0	0.50	0	0
NRP	Diisonitrile antibiotic SF2768	0.66	0.66	0.61	0.66	0.61
	Scabichelin	1.00	0	0.90	1.00	0
	ε-Poly-L-lysine	0	1.00	1.00	1.00	1.00
	Cysteoamide	0	0	0.63	0.63	0
	Peucechelin	0	0	0.25	0	0
	Thaxtomin A	0	0	0	0.81	0
	Rotihibin A	0	0	0	0	1.00
NRP+Polyketide:Modular type I polyketide	Tirandamycin	0	0.53	0	0	0
NRP+saccharide	Minimycin	0	0	1.00	1.00	0
Other	γ-butyrolactone	1.00	1.00	1.00	1.00	1.00
	Ectoine	1.00	1.00	1.00	1.00	1.00
	EDHA	0.88	0	0	0	0
	Triacsin C	0.65	0	0	0	0
	Desferrioxamin B/desferrioxamine E	0	1.00	0.83	1.00	1.00
	Melanin	0	0	0	1.00	0.60
Polyketide	Spore pigment	0.83	0.83	0.75	0.58	0.83
	Inthomycin B	0.54	0	0	0	0
	Alkylresorcinol	0	1.00	0	0	0
	Viguiepinol	0	0	0.53	0	0
	A-74528	0	0	0	0.55	0
	Tripartilactam/niizalactam C	0	0	0	0	0.64
Polyketide:Type II polyketide	Fredericamycin A	0	0	0.81	0	0.81
Polyketide+other	Tetrafibricin	0	1.00	0	0	0
Ripp	Humidimycin	0.92	0	0	0	0
Ripp:Lanthipeptide	Informatipeptin	1.00	1.00	1.00	0	0
	Streptocollin	0	0	0	0	1.00
Terpene	Hopene	0.84	0.92	0.92	0.92	0.92
	Albaflavenone	1.00	1.00	1.00	1.00	1.00
	Avermitilol	0	1.00	0	0	0
	2-methylisoborneol	0	0	1.00	1.00	0

1- BCCO 10_0861^T^, 2- *S. lacrimifluminis* DSM 111125^T^, 3- *S. reticuliscabiei* DSM 41804^T^, 4- *S. turgidiscabies* DSM 41838^T^, 5- *S. geranii* JCM 32177^T^

The key differences in AMR mechanisms among the BCCO 10_0861^T^ and closest relatives is the lack of aminoglycoside antibiotic inactivation enzyme (AAC(2')-I), and antibiotic target replacement protein 3-hydroxyacyl-thioester dehydratase (HtdX). In addition, BCCO 10_0861^T^ lack tetracycline antibiotic target protection (Tet) in comparison with *S. geranii* JCM 32177^T^, *S. reticuliscabiei* DSM 41804^T^ and *S. turgidiscabies* DSM 41838^T^. BCCO 10_0861^T^ shares multiple transferable resistance A (MtrA) multiple transferable resistance B (MtrB), oxidative stress response regulator (OxyR) as regulators and protein altering cell wall structure (VanJ) with all other strains except *S. geranii* (Supplementary Table 6). MGE analysis in five *Streptomyces* species reveals that BCCO 10_0861^T^ did not have any detectable MGEs. Alternatively, *S. geranii*, *S. reticuliscabiei*, *S. lacrimifluminis*, and *S. turgidiscabies* contain various insertion sequences (IS) such as ISSgr2, ISSdi1, ISMysp4, ISStac1, ISBfu1, IS469, ISSam1, ISStu2, IS112, and ISSav6, with *S. reticuliscabiei* being the sole one to carry a composite transposon (cn_4078_ISBfu1) (Supplementary Table 6).

Pan-genome analysis revealed that BCCO 10_0861^T^ possesses the highest number of orthologous genes (10,827 according to the Database of Clusters of Orthologous Genes, COGs), followed by *S. turgidiscabies* with 10,318, *S. reticuliscabiei* with 9,632, *S. geranii* with 9,258, and *S. lacrimifluminis* with 8,587. These strains share 4,190 identical genes, while BCCO 10_0861^T^ exhibits 4,287 unique genes, as shown in Supplementary Table 7 with an accompanying Venn diagram.

## Chemotaxonomic characterization

Strain BCCO 10_0861^T^ was incubated in ISP2 medium for chemotaxonomic analysis: a preculture grown in 20 mL for 48 h (150 rpm, 28 °C) served as inoculum for 100 mL main culture, which was grown for 48 h at 150 rpm and 28 °C. Biomass was collected by centrifugation (1,000 × *g* for 10 min) and freeze-dried (L10-55 PRO, GREGOR Instruments, s.r.o., Říčany, Czech Republic) before further usage (Lara et al. 2024).

Freeze-dried biomass was used for the detection of whole-cell sugar composition (Lechevalier and Lechevalier 1970), polar lipids (Minnikin et al. 1984), and diaminopimelic acid (Staneck and Roberts 1974) detected by thin-layer chromatography (TLC). The cellular fatty acid analysis was performed by gas chromatography–flame ionization detector (GC-FID) followed by GC-mass spectrometry (GC-MS) run on an Agilent GC–MS 7000D instrument as described in Sasser 2001; Vieira et al. 2021. High-performance liquid chromatography (HPLC) coupled with a diode array detector (DAD) and a high-resolution mass spectrometer was used for isoprenoid quinone detection (Schumann et al. 2021).

Whole cell hydrolysates showed the presence of *LL*-DAP as the diagnostic diamino acid for the peptidoglycan, which agreed with the selected strains included in the comparison. The composition of sugars in whole cell hydrolysates and phospholipid constituents differed significantly between strain BCCO 10_0861^T^ and the most related strains (Table 2). In BCCO 10_0861^T^ galactose, glucose, mannose, ribose, and traces of rhamnose were present as whole cell sugars, while arabinose and xylose were additionally present in *S*. *reticuliscabiei* DSM 41804^T^ and *S*. *turgidiscabies* DSM 41838^T^. On the other hand, only galactose or galactose together with ribose were present in whole cell hydrolysates in *S. lacrimifluminis* DSM 111125^T^ and *S. geranii* JCM 32177^T^, respectively. Chemotaxonomic analysis of strain BCCO 10_0861^T^ revealed a menaquinone profile consisting of MK-9 (H_6_) (57.6%), MK-9 (H_8_) (19.6%), MK-9 (H_4_) (14.0%), and MK-9 (H_2_) (8.8%), being the most similar to the menaquinone composition of *S. reticuliscabiei* DSM 41804^T^. Contrastingly, MK-9 (H_8_) was of elevated abundance in strains *S. lacrimifluminis* DSM 111125^T^ and *S. geranii* JCM 32177^T^, reaching 48.1% and 38.4%, respectively.

Polar lipid pattern composed of aminophospholipid (APL), diphosphatidylglycerol (DPG), glycolipids (GPL), phosphatidylethanolamine (PE), phosphatidylinositol (PI), and phospholipids (PL) (Supplementary Fig 3). Major cellular fatty acids (> 5%) included C_16:0_ (19.5%), *anteiso*-C_15:0_ (15.6%), *iso*-C_16:0_ (15.2%), and C_16:1_ ω7c (14.3%), *iso*-C_15:0_ (8.2%), and *anteiso*-C_17:0_ (6.4%) (Table 2**)**. The composition of fatty acids of strain *S*. *lacrimifluminis* DSM 111125^T^, *S*. *reticuliscabiei* DSM 41804^T^ and *S*. *turgidiscabies* DSM 41838^T^ differed from those of BCCO 10_0861^T^ only by the relative abundance, while it significantly differed from strain *S*. *geranii* JCM 32177^T^, in which *iso*-C15:0 and C16:1 ω7c were completely absent. More details on the fatty acid compositions are given in Supplementary Table 8. The chemotaxonomic features of the strain were similar to its close neighbors *S. lacrimifluminis* DSM 111125^ T^, *S. reticuliscabiei* DSM 41804^ T^, and *S. turgidiscabies* DSM 41838^ T^ (Table 2).

## Antimicrobial activities

*Streptomyces* sp. strains were inoculated on Mueller–Hinton agar (Sigma-Aldrich, Co., St. Louis, MO), four days in advance and cultivated at 28 °C. The overnight cultures (16 h) of indicator strains (37 °C) were added to streptomycetes in a T-shape and co-cultivated at 28 °C to next day for reading the results (Herbrík et al. 2020). Strain BCCO 10_0861^T^ showed different pattern of inhibitory activities compared to other species. It weakly inhibited the growth of *Bacillus subtilis* CCM 1718^T^ and *Moraxella catarrhalis* (clinical isolate), inhibited *Candida albicans* CCM 8186^T^, however did not affect the growth of *Staphylococcus aureus* DSM 346^T^, *Escherichia coli* DSM 682^T^, *Klebsiella pneumoniae* DSM 681^T^, and *Pseudomonas aeruginosa* DSM 50071^T^ (Table 4).

**Table 4:** Spectrum of antimicrobial activities of *S. chodovensis* sp. nov. = BCCO 10_0861^T^ strain and closest relatives against Gram-positives, Gram-negatives, and fungi

	1	2	3	4
*Bacillus subtilis* CCM 1718^ T^	+	+++	−	−
*Staphylococcus aureus* DSM 346^ T^	−	−	−	−
*Klebsiella pneumoniae* DSM 681^ T^	−	−	−	−
*Moraxella catarrhalis* (clinical isolate)	+	+++	+	+++
*Pseudomonas aeruginosa* DSM 50071^ T^	−	−	−	−
*Acinetobacter baumannii* (clinical isolate H4921)	−	−	−	−
*Candida albicans* CCM 8186^ T^	++	+++ (+ ++ +)*	++	++

Inhibitory activity categories according to observed zone size:—no inhibition; + 0.1–0.5 cm; ++ 0.6–1.0 cm; +++ 1.1–3.0 cm. *Extended pre-cultivation of *Streptomyces* strain resulted in detectable inhibition zones (7–10 days). 1- BCCO 10_0861^T^, 2- *S. lacrimifluminis* DSM 111125^T^, 3- *S. reticuliscabiei* DSM 41804^T^, 4- *S. turgidiscabies* DSM 41838^T^

## Plant growth promoting traits

Plant growth promoting traits were detected with PlaBA-db (Patz et al. 2021). Examination of plant growth-promoting traits (PGPT)-associated genes in the genomes indicated that BCCO 10_0861^T^ has the most abundant gene count at 4,811, followed by *S. geranii* with 4,289, *S. turgidiscabies* with 4,401, *S. reticuliscabiei* with 4,319, and *S. lacrimifluminis* with 3,844. They collectively share 885 identical PGPT-associated genes, yet BCCO 10_0861^T^ contains 2,255 distinct genes compared to the others, as presented in Table 1 and Supplementary Table 9 with a corresponding Venn diagram.

To investigate plant growth-promoting traits, experiments were conducted with a minimum of two technical replicates (Dede and Güven 2023). Actinomycetes in their active growth phase were cultured on Pikovskaya agar (PA) (Pikovskaya 1948) to assess phosphate solubilization (PS). For indole-3-acetic acid (IAA) production, endophytic bacteria were inoculated in peptone water medium and incubated at 28 ± 2 °C for 28 days on a rotary shaker at 120 rpm (Gordon and Weber 1951). The culture was then centrifuged at 720 g for 30 min, and 0.5 mL of the supernatant was mixed with 1 mL of Salkowski reagent. After 30 min in the dark at room temperature, a pink coloration indicated positive IAA production. Additionally, IAA production was measured spectrophotometrically at 530 nm with a Biotek Epoch2 microplate reader. Tryptophan-dependent IAA production was evaluated by supplementing peptone water with 0.2% DL-tryptophan (Merck) and incubating at 28 ± 2 °C for 7 days (Dede et al. 2020). Hydrocyanic acid (HCN) production was assessed on modified ISP2 agar supplemented with glycine (4.4 g/L) under anaerobic condition (Lorck 1948; Dede and Güven 2024). Data are provided in Supplementary Table 10. For siderophore production, actinomycetes were grown on modified chrome azurol S (CAS) agar (Schwyn and Neilands 1987) (blue dye and NA mixture) consisting of a blue dye and nutrient agar mixture, and incubated at 28 ± 2 °C for 3 days, following the protocol by Dede et al. (2020).

Siderophore production assays confirmed the presence of desferrioxamine-type siderophores in *S. lacrimifluminis*, *S. reticuliscabiei*, and *S. turgidiscabies* (Table 3). However, strain BCCO 10_0861^T^ did not produce siderophores in the assay, despite genomic predictions suggesting the potential for scabichelin-type siderophore production, which was not experimentally validated. In contrast, strain BCCO 10_0861^T^ demonstrated the ability to produce soluble proteases, IAA, and solubilized phosphates from tricalcium phosphate. Notably, strain BCCO 10_0861^T^ was the only strain that produced hydrocyanic acid. The findings are presented in Table 5.

**Table 5:** Spectrum of plant growth promoting traits of *S. chodovensis* sp. nov. BCCO 10_0861^T^ strain and closest relatives

	1	2	3	4
Siderophore production	−	+ (1 mm)	+ (7 mm)	+ (7 mm)
Phosphate solubilization	+ (1.5 mm)	+ (4 mm)	+ (15 mm)	+ (9 mm)
Protease production	+ (1 mm)	+ (1.5 mm)	+ (1 mm)	+ (1 mm)
HCN production	+	−	−	−
IAA production	+	+	+	+

—Refers to no activity or zone observed; + refers to positive response. The mean zone size is given in the brackets, when indicated. 1- BCCO 10_0861^T^, 2- *S. lacrimifluminis* DSM 111125^ T^, 3- *S. reticuliscabiei* DSM 41804^ T^, 4- *S. turgidiscabies* DSM 41838^ T^

## Description of *Streptomyces chodovensis* sp. nov.

*Streptomyces chodovensis* (cho.do.ven.sis, L. masc. adj. *chodovensis*, of Chodov, named after the place of origin of type strain, the town Chodov, Czech Republic).

Gram-positive bacterium. Substrate mycelium is pink to brown on ISP2, ISP3, ISP4 and ISP7. Dense, white to gray aerial mycelium is produced on ISP2, ISP4 and ISP7. Produces pink-orange soluble pigment on ISP2, ISP3, ISP4 and ISP7. Melanin pigment is not produced on either ISP6 or ISP7. Sporophores are of rectus flexibilis shape, spores are rod-shaped 1.5 µm long and 0.7 µm wide (median values). Grows at 4–30 °C with optimal growth at 28 °C. Grows at pH 6–8. The strain can easily utilize glycerol, D-mannose, and D-xylose; can utilize D-glucose, D-mannitol, I-inositol, L-rhamnose, and sucrose; shows weak utilization of L-arabinose; and cannot utilize cellulose, D-fructose, or D-ribose.

The strain tested positive for acid phosphatase, alkaline phosphatase, α-galactosidase, α-glucosidase, β-galactosidase, β-glucosidase, cystine arylamidase, esterase, esterase lipase, leucine arylamidase, naphthol-AS-BI-phosphohydrolase, N-acetyl-β-glucosaminidase, trypsin, and valine arylamidase; while showing weak activity for α-chymotrypsin, α-fucosidase, α-mannosidase, β-glucuronidase, and lipase. The cell wall is of the III type (*LL*-DAP). The whole cell sugar pattern consists of galactose, glucose, mannose, ribose, and traces of rhamnose. Phospholipid pattern consists of DPG, PE, PLs, APL, PI, and GPL. The predominant menaquinone is MK-9 (H_6_). The major fatty acids (> 5%) are C_16:0_, *anteiso*-C_15:0_, *iso*-C_16:0_, and C_16:1_ ω7c, *anteiso*-C_17:0_, *iso*-C_15:0_. The G+C content of the DNA is 70.05%.

The type strain, BCCO 10_0861^T ^= DSM 117345^T^, was isolated from a bare soil sample in Sokolov Coal Basin, Czech Republic. The GenBank accession number for the 16S rRNA gene sequence of strain BCCO 10_0861^T^ is OR827066. The whole genome shotgun project has been deposited in European Nucleotide Archive (ENA) under accession number Project No. PRJEB70906.

## Supplementary Information

Below is the link to the electronic supplementary material.

Supplementary file1 (PPTX 3673 KB)

Supplementary file2 (XLSX 1742 KB)

## Data Availability

All data generated in this study were deposited into the ASEP Repository and are available under https://doi.org/10.57680/asep.0644366. The reads and assembly were deposited at the ENA under the study accession ERP155806, sample accession ERS17732811, reads accession ERR12495476, and assembly accession ERZ22297350. The GenBank accession number for the 16S rRNA gene sequence is OR827066.
